# Fabrication of Biomimetic Cell Culture Membranes Using Robust and Reusable Nickel Micropillar Molds

**DOI:** 10.1007/s13206-024-00179-7

**Published:** 2024-12-10

**Authors:** Taiki Otomo, Hyunsoo Noh, Tatsuya Matsubara, Deok-Ho Kim, Masashi Ikeuchi, Kazuhiro Yoshida, Joon-wan Kim

**Affiliations:** 1 Department of Mechanical Engineering, Tokyo Institute of Technology, J3-12, 4259 Nagatsuta-Cho, Midori-Ku, Yokohama, 226-8503 Japan; 2 Department of Biomedical Engineering, Center for Microphysiological Systems, Johns Hopkins University, 720 Rutland Avenue, Baltimore, MD 21205 USA; 3 Department of Biomedical Engineering, Department of Medicine, Department of Mechanical Engineering, Center for Microphysiological Systems, Institute for NanoBio Technology, Johns Hopkins University, 720 Rutland Avenue, Baltimore, MD 21205 USA; 4 Institute of Biomaterials and Bioengineering, Tokyo Medical and Dental University, Building No.22, 2-3-10 Surugadai, Kanda, Chiyoda-Ku, Tokyo Japan; 5 Laboratory for Future Interdisciplinary Research of Science and Technology (FIRST), Institute of Innovative Research (IIR), Tokyo Institute of Technology, J3-12, 4259 Nagatsuta-Cho, Midori-Ku, Yokohama, 226-8503 Japan

**Keywords:** Microfabrication, Organ-on-a-chip, Porous membrane, Biomimetic membrane

## Abstract

In the practical application of organ-on-a-chip, mass production technology for flexible porous membranes is an essential element for mimicking the basement membrane of the body. Porous PDMS membrane is a promising material due to its high optical transparency, flexibility, and biocompatibility. However, the fabrication process is complex and costly. Even with soft lithography, a relatively straightforward method, there is a risk that the negative resist pillars used as molds peeling off from the substrate in mass production. In this study, we propose a novel mass production method for fabricating porous PDMS membranes using high-strength nickel (Ni) micropillars as molds by combining photolithography and electroforming technologies. The unibody structure of Ni micropillars ensures high reliability and provides a semi-permanent mold without degradation or detachment. We successfully fabricated two types of Ni micropillars and subsequently formed their corresponding porous PDMS membranes (*D* (diameter) = 8 μm, *P* (pitch) = 30 μm, and *D* = 10 μm, *P* = 20 μm). The porous PDMS membrane showed non-inferiority to the control group in terms of viability when cultured with human vascular endothelial cells. Furthermore, we showed that the porous PDMS membrane can be used to evaluate the vascular permeability of nanoparticles.

## Introduction

Organ-on-a-chip (OOC) and other biomimetic systems are expected to be applied in drug discovery and biological research, owing to their potential to model diseases and reproduce cellular environments with high reproducibility. OOC is a miniaturized in vitro model that can replicate the functions of organs and cell–cell interactions in a human organism by culturing cells on a microfluidic device [1–3]. Previously, various devices have been developed to mimic various organs such as the eye [4], lungs [5, 6], blood vessels [7], heart [8], and liver [9]. It has been reported that a dynamic environment similar to that of in vivo experiments can be reproduced in vitro by stretching alveolar and myocardial tissues cultured on flexible membranes [5, 10].

One of the most important components of the OOC is the culture membrane, which serves as the substrate for organizing the cellular tissues [11]. To accurately reproduce the in vivo environment, membranes must mimic the extracellular matrix (ECM), the porous structure that supports in vivo cellular tissue. The ECM's porosity facilitates the exchange and interaction of nutrients between tissues [12]. Its flexibility also enables the deformation of biological tissues. Mimicking both the porosity and flexibility of the ECM is essential to reproduce the dynamic in vivo environment [12].

Conventional OOC systems utilizes membranes made of polycarbonates (PC) or polyethylene terephthalate (PET) [13, 14], membranes composed of nanofibers [15, 16], or porous membranes made of polydimethylsiloxane (PDMS) [1, 5]. PC and PET are biocompatible materials commonly used in static culture systems [17, 18]. However, their lack of flexibility prevents these membranes from being integrated into devices that require stretching to provide mechanical stimulation. Additionally, their low transparency may interfere with the optical evaluation of cellular properties [19]. Membranes formed from nanofibers exhibit a porous structure similar to the in vivo ECM [20, 21]. Notably, nanofiber culture membranes made of polylactic acid (PLA) and polylactic glycolic acid (PLGA) are highly biocompatible and have been used to culture cancer cells in microdevices, as well as serving as culture membranes for lung-on-a-chip [15, 22]. However, there are concerns that cells may be cultured non-uniformly due to the lack of precise control over the pore size, pore spacing (pitch), and fiber orientation [23, 24]. Similarly to PC membranes, the low transparency of nanofiber membranes also poses challenges for their application as culture membranes in devices [11]. To overcome the problems related to optical transparency and flexibility, many researchers have focused on PDMS [25]. Widely used in the field of microfluidic devices, PDMS is particularly suitable for producing porous membranes due to its ease of microfabrication through soft lithography and replica molding[26]. It has potential applications not only as a scaffold for cells but also as a filter to be integrated into cell sorting devices [27, 28]. Consequently, porous culture membranes made of PDMS have been successfully applied in devices that provide mechanical stimulation to cells [1, 5, 6].

Previous porous PDMS membrane fabrication methods can be divided into two types: dry etching and soft lithography. Dry etching involves selectively etching PDMS through a metal mask with a porous pattern [25], producing small pores (pore diameter: 2 μm) without requiring molds. However, this technique is complex, costly, and time-consuming for each membrane fabrication. In contrast, soft lithography is a simpler and more cost-effective method. It uses micropillars as molds to create porous membranes, with several methods available for fabricating these pillars. One method, proposed by Zakharova [29], uses positive resist to form pillars that dissolve after casting the PDMS, eliminating the need to release the membranes from the pillars and reducing the risk of membrane damage. However, forming micropillars for each membrane is a one-time use process and labor-intensive. Another method uses negative photoresist, SU-8 [30, 31], which is also simple if a photolithography environment is available. However, there is a risk that the micropillars may also detach from the substrate when the PDMS membrane is peeled off. Keshtiban [31] overcame this problem by applying a PVA coating as a sacrificial layer on the mold to protect both the mold and the membrane, though the dissolution and device assembly processes remain challenging. Additionally, a method using silicon (Si) wafers, etched by deep reactive ion etching (DRIE) has been proposed [32, 33]. While Si pillars are robust and reusable, their formation is expensive and time-consuming [32]. Lastly, for forming penetrated pores, a punching technique is applied to exert pressure on the PDMS-coated micropillar [34, 35]. This technique, however, is technically challenging due to the need to adjust the punching pressure, necessitating a simpler operation. Thus, conventional membrane fabrication in microfluidics is inefficient and time-consuming. Therefore, further research is needed on new fabrication techniques for PDMS porous membranes in microfluidics [26].

In this study, we propose a novel method for fabricating porous PDMS membranes using nickel (Ni) micropillars formed by Micro-Electro-Mechanical System (MEMS) technology. The Ni micropillars are integrated directly with a metal substrate, enhancing the structural integrity of the pillars and enabling their repeated use without risk of detachment. This integration results in significantly stronger adhesion between Ni and the metal substrate (483 MPa [36]), compared to the adhesion between SU-8 and a Si substrate (20.7 MPa [37]). Although the fabrication process of these robust micropillars is more demanding than that of SU-8 pillars, their durability makes them well-suited for large-scale production without requiring advanced equipment like deep reactive ion etching (DRIE). We further propose modifying the pillar surface to be hydrophobic, which allows the casting of porous PDMS membranes solely by the spin-coating process. To the best of our knowledge, this is the first application of electroformed Ni micropillars as molds in the fabrication of porous PDMS membranes. Our study not only demonstrates the effectiveness of this method but also confirms its potential as a viable manufacturing process for organ-on-a-chip (OOC) applications, as shown by successful cell cultures on the membranes integrated into culture inserts.

## Materials and Method

### Design and Fabrication of Ni Micropillar Molds

We propose fabricating Ni micropillars using photolithography and electroforming techniques. In this study, two types of Ni micropillars are fabricated to demonstrate the versatility of the processing method, with dimensions of *D* (diameter) = 8 μm, *P* (pitch) = 30 μm, and *D* = 10 μm, *P* = 20 μm. These dimensions are based on those commonly used in conventional culture inserts and organ-on-a-chip systems [5, 38]. To achieve a membrane thickness similar to that of conventional PDMS membranes, which is less than 10 μm [5, 6], the pillar height is set to 15 μm, exceeding the membrane thickness. Consequently, the aspect ratios (diameter: height) of the micropillars are established as 10:15 and 8:15.

**Fig. 1:**
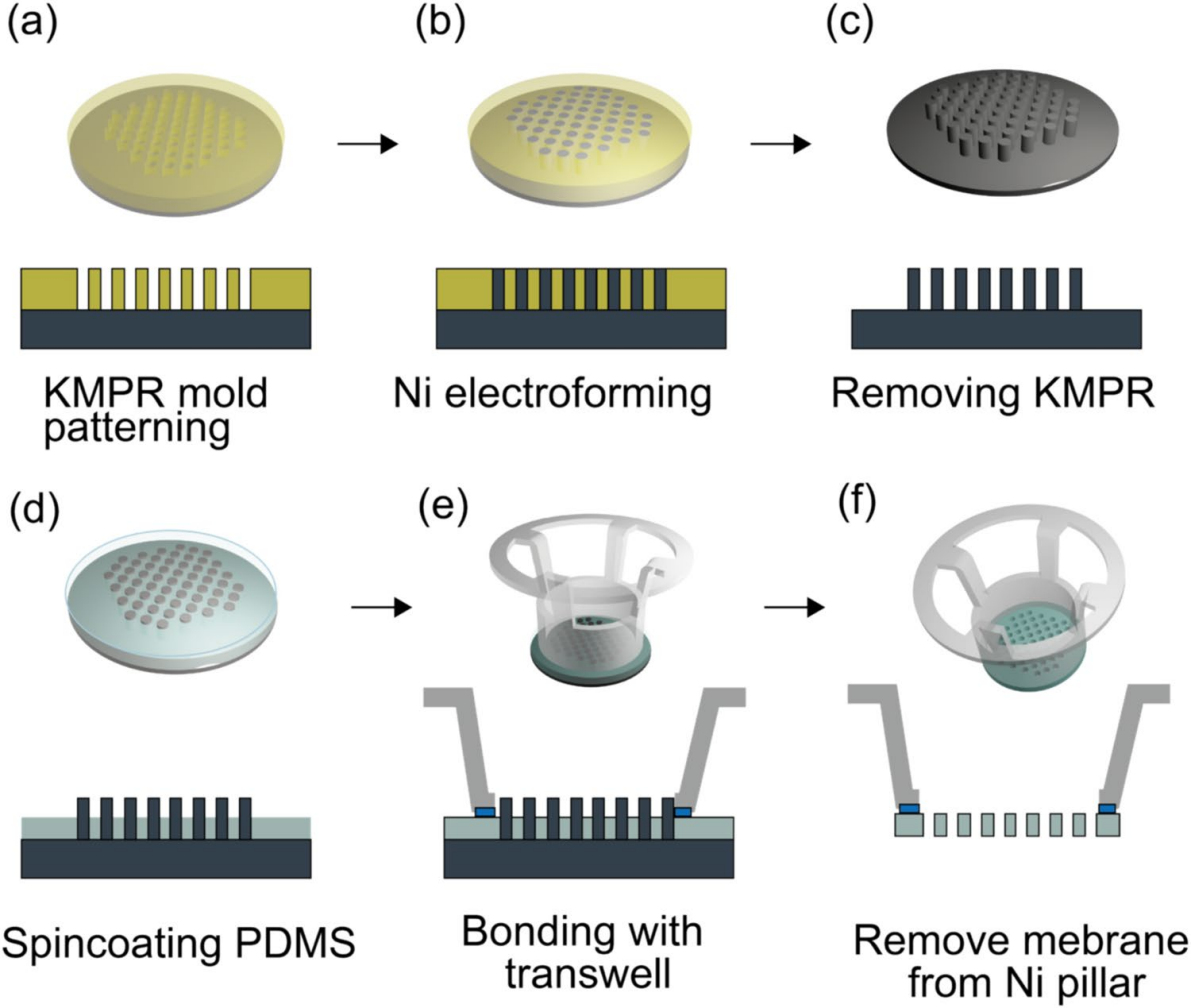
Fabrication and assembly process of the microporous membrane and cell culture insert. **a** Patterning the mold using negative photoresist for Ni electroforming. **b** Fabricating Ni micropillars by electroforming. **c** Removing the negative photoresist. **d** Spin-coating the PDMS precursor onto the Ni micropillars. **e** Bonding the cell culture inserts with the spin-coated PDMS. **f** Releasing the PDMS with bonded culture insert from the Ni micropillars

First, the surface of a 30 mm diameter stainless steel disc (TRUSCO NAKAYAMA CORPORATION) is polished using PiKAL (NIHON MARYO KOGYO CO., LTD.) to prevent diffuse reflection during photolithography. The entire surface of the polished disc is then electroplated with nickel. Subsequently, a thick layer of negative photoresist (KMPR 1035, MicroChem Corp.) is spin-coated at 4000 rpm for 40 s and soft-baked at 100°C for 7 min. UV light is selectively exposed using a Desktop Maskless Lithography System (PALET, NEOARK Corporation) to pattern the micromold (Fig. 1a). Next, Ni micropillars are formed in the photoresist mold by electroforming (Fig. 1b). The Ni micropillar growth proceeds from the seed layer at the bottom of the mold upwards by electroforming, continuing until reaching the desired mold height. Finally, the photoresist mold is removed using Remover K and Neutralizer K (MicroChem Corp.), which ensures complete dissolution of the photoresist without damaging the Ni micropillars (Fig. 1c).

**Fig. 2:**
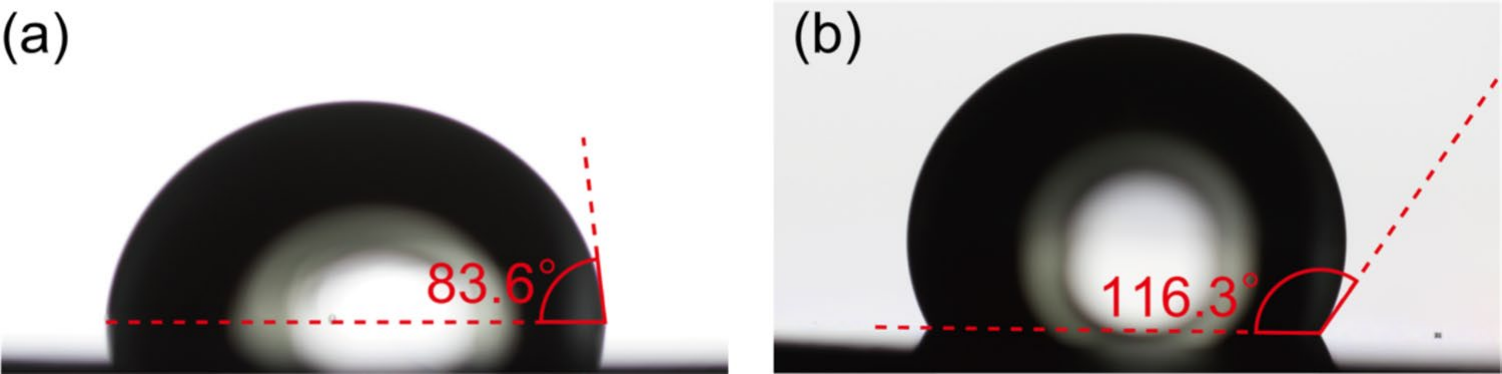
Contact angles of the Ni-plated surface. **a** Before hydrophobic treatment. **b** After application of a fluoride coating agent for hydrophobic treatment

When spin-coating the PDMS precursor onto the micropillars, it may cover the top of the micropillars and prevent the formation of a completely penetrated pore [39]. Additionally, another issue is the difficulty in peeling the PDMS membrane from the mold, which can lead to membrane destruction. Both of these problems can be addressed by one method: hydrophobic treatment of the mold surface [30]. A hydrophobic surface treatment is applied to the micropillars to prevent the PDMS precursor from climbing to the top surface of the micropillar. Moreover, the hydrophobic surface of the mold facilitates the detachment of the cast PDMS membrane [34]. The treatment involves spin-coating a fluoride coating agent (FS-1060TH, Fluoro Technology Co., LTD.) at 1000 rpm for 60 s. To enhance the adhesion of the coating agent, it is baked at 100 °C for 30 min. Figure 2a and b show the contact angles of the plate surfaces before and after hydrophobic coating.

### PDMS Molding Procedure

Porous PDMS membranes are cast by spin-coating the precursor onto a substrate with micropillars. PDMS (Sylgard 184, Dow Corning Toray Co., Ltd.) is mixed with a curing agent at a weight ratio of 10:1. The prepolymer is then diluted with toluene to decrease its viscosity (weight ratio 1:1), enabling a uniform coating of PDMS that is thinner than the height of the pillars. The diluted PDMS prepolymer is then spin-coated onto the Ni micropillars at 4000 rpm for 120 s (Fig. 1d). The coated PDMS is left at room temperature for 30 min to smoothen the surface and then cured on a hot plate at 90°C for 1 h. For cell culture applications, the polycarbonate (PC) membrane at the bottom of the culture insert (ThinCert, Greiner Bio-One) is removed and replaced with the fabricated PDMS membrane. The PDMS adhesive (pre-polymer: curing agent, 10:1 weight ratio) is applied to the culture insert using a disposable scalpel. The culture inserts and the porous membranes cast in the mold are bonded using PDMS adhesive (Fig. 1e). These are heated on a hot plate at 60°C for 3 h to cure the PDMS adhesive. The bonded porous membrane and culture insert are then released from the mold, forming a culture unit containing the culture insert and porous PDMS membrane (Fig. 1f).

## Results and Discussion

### Ni Micropillar Fabrication

**Fig. 3:**
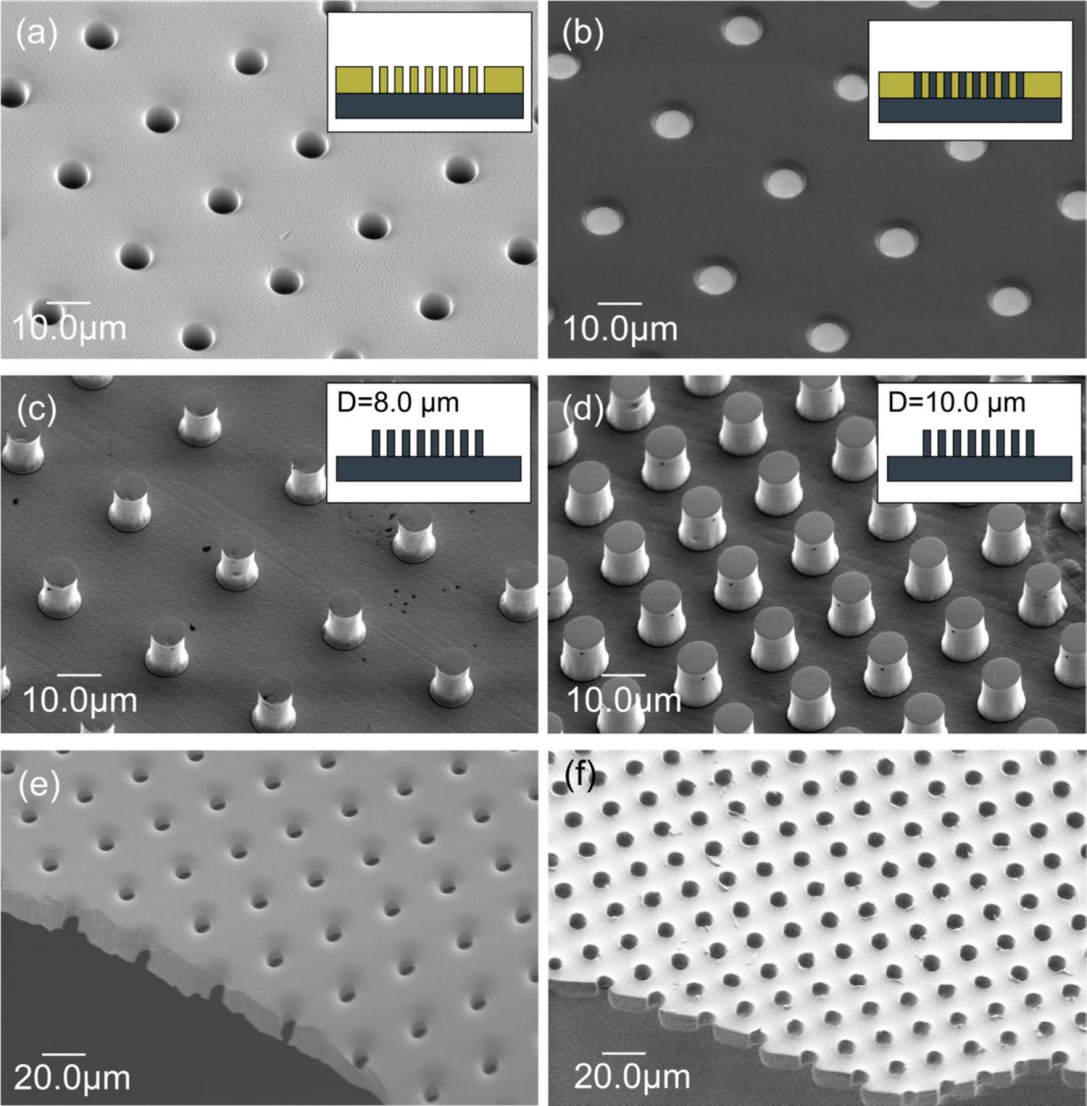
Fabrication of Ni micropillar mold by MEMS technology. **a** Patterned KMPR mold for electroforming. **b** KMPR mold after the electroforming process. **c** Fabricated Ni micropillars after removing KMPR mold with dimensions *D* = 8 μm, *P* = 30 μm, aspect ratio is 8:15, **d** Fabricated Ni micropillars with dimensions of *D* = 10 μm, *P* = 20 μm. aspect ratio is 10:15, **e** Cross-sectional image of fabricated porous PDMS membrane with dimensions *D* = 8 μm, *P* = 30 μm; and (f) *D* = 10 μm, *P* = 20 μm

Patterning of negative resist molds with dimensions of 8 µm in diameter and 30 µm pitch was successfully fabricated, as shown in Fig. 3a. The dimensions of the patterned pores were measured from the SEM images using ImageJ. The average pore size formed in the mold was approximately 8.1 μm, indicating that the patterning closely matched the designed values. It was observed that the Ni micropillars grew to the same height as the mold (Fig. 3b). Upon removal of the negative resist, it was confirmed that the Ni micropillars were properly formed without any issues (Fig. 3c). Although the lower part of the pillars was observed to be larger than the upper part, this discrepancy did not significantly affect the membrane formation. Similarly, pillars with a diameter of 10 µm and a pitch of 20 µm were successfully formed using the same process (Fig. 3d).

### Porous PDMS Membrane Fabrication

**Fig. 4:**
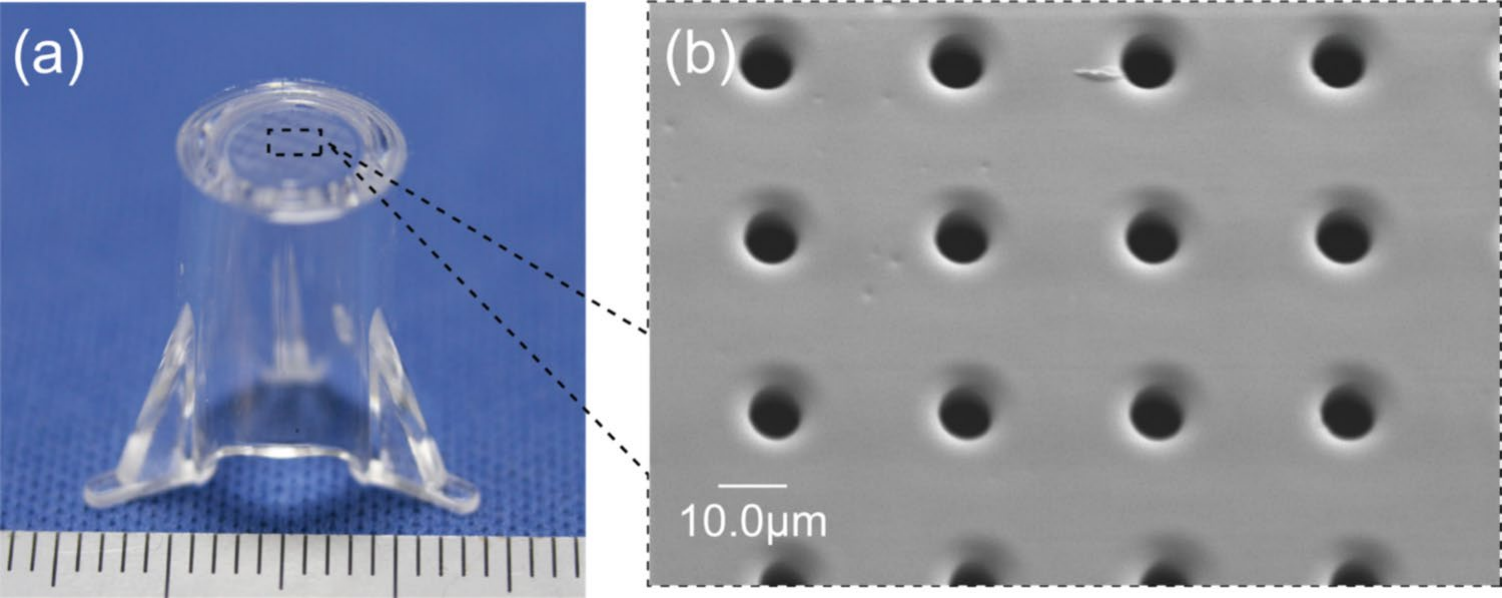
Fabrication of the cell culturing unit with a porous PDMS membrane, released from the Ni micropillars after bonding the culture insert frame and the porous PDMS membrane. **a** Assembled image of the cell culturing unit. **b** Close-up view of the fabricated membrane with dimensions *D* = 8 μm, *P* = 30 μm

Figure 3e and f display SEM images of the PDMS membrane released from the Ni micropillar molds. The diameters of the pores were measured to be approximately 7.96 µm and 9.15 µm, which are about 10% smaller than the designed values, demonstrating the feasibility of forming pillars close to the required dimensions. The SEM image of the cross-section of the membrane confirms that the pores completely penetrated each membrane, indicating successful formation of a porous membrane. The porous membrane was then bonded to the culture insert and successfully assembled, as shown in Fig. 4a and b.

### Culturing Cells on Porous PDMS Membrane

**Fig. 5:**
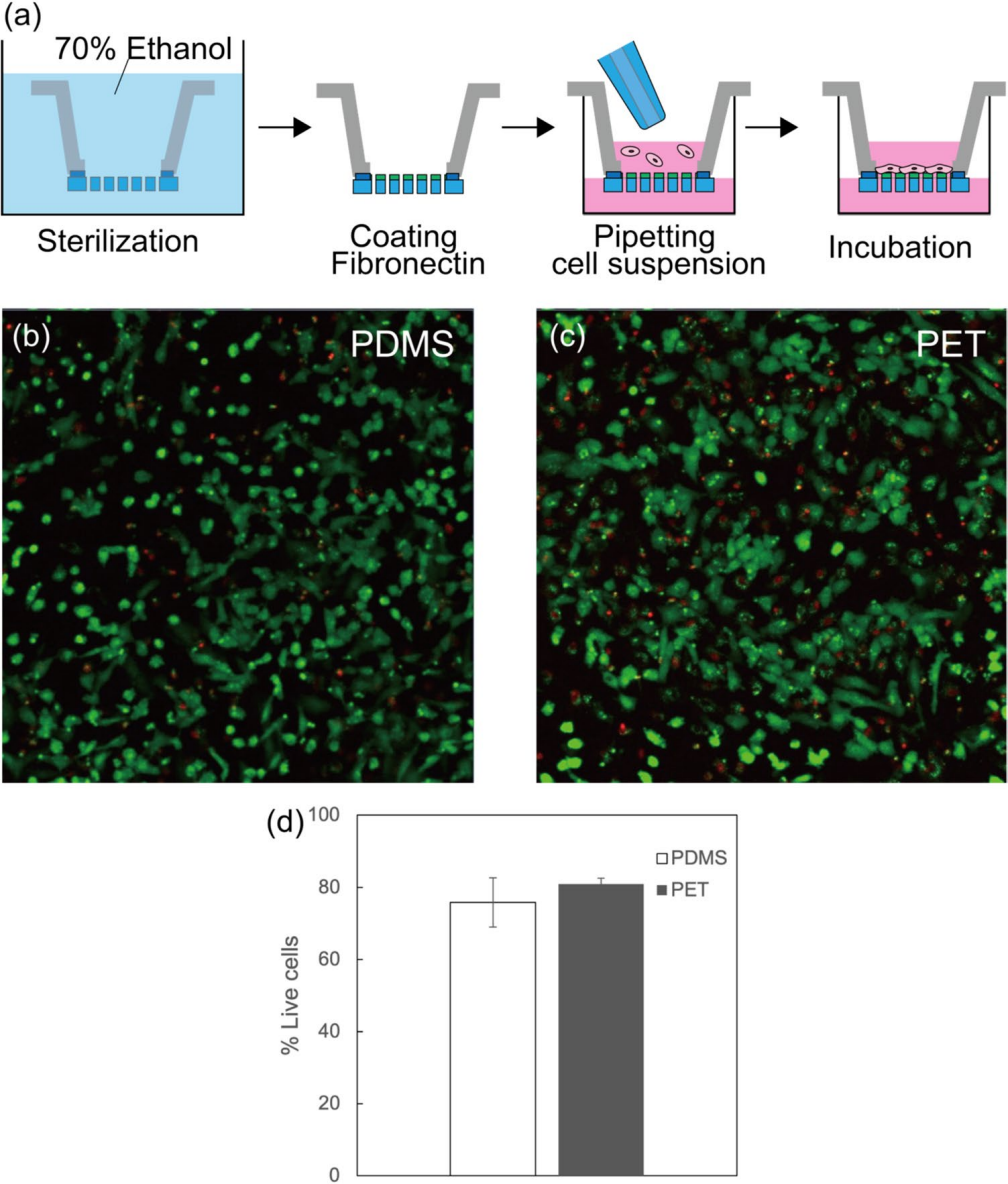
Viability evaluation of the porous PDMS membrane. **a** Procedure of cell culturing on the PDMS membrane. **b** Live/dead staining of HUVECs cultured on the PDMS membrane and **c** on the conventional PET membrane. **d** Quantitative analysis by counting live cells

Human umbilical vein endothelial cells (HUVECs) were cultured on our fabricated PDMS membranes with dimensions *D* = 8 μm, *P* = 30 μm to evaluate their viability as culture membranes. The culturing process on the membrane as shown in Fig. 5a was performed using conventional methods [29]. First, the culture unit, consisting of the cell culture insert frame and the porous PDMS membrane, was immersed in 70% ethanol for 1 h to sterilize it. The sterilized membrane was then washed twice with phosphate-buffered saline (PBS, FUJIFILM Wako Pure Chemical Corporation) to completely remove any residual ethanol. The membrane was coated with human plasma fibronectin (Sigma-Aldrich) diluted in PBS to 5 μg/mL to improve cell adhesion on both the PDMS membrane and the conventional culture insert. HUVECs obtained from confluent flasks were plated onto the fibronectin-coated membrane at a concentration of 2.0 × 10^6^ cells/mL (4.0 × 10^5^ cells). The HUVECs were incubated in Humedia-EG2 (Kurabo Industries Ltd.) at 37°C and 5% CO_2_.

To confirm whether dilution of PDMS with toluene is toxic to cells, HUVEC viability was assessed using a live/dead assay. Live and dead cells were stained 48 h after cell seeding using the LIVE/DEAD® Viability/Cytotoxicity Kit (Thermo Fisher). Stained cells were observed with an inverted confocal microscope, LSM780 (ZEISS). The ratio of viable cells was compared between the fabricated PDMS membrane and the conventional PET one. As shown in Fig. 5b and c, cells were stained using the live/dead assay. The number of viable (green) and dead (red) cells was analyzed using ImageJ to determine the ratio of viable cells to the total cell count. HUVEC exhibited sufficient adhesion to each membrane type, with viability ratios of 75.8% for the PDMS membrane and 80.9% for the PET membrane, indicating that the dilution of toluene was not toxic to HUVECs. This result suggests that the porous PDMS membranes fabricated by the proposed method in this study can function effectively as culture membranes.

### Permeability Assay

**Fig. 6:**
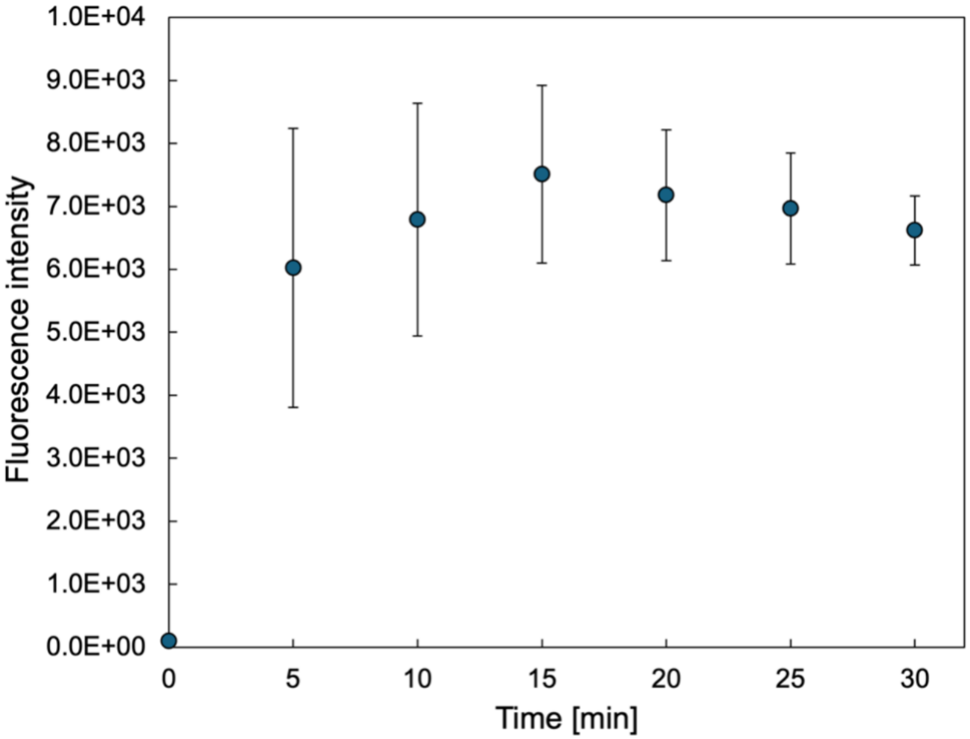
Permeability evaluation of the cultured PDMS porous membrane

Porous membranes are expected to facilitate substance exchange through cells. To evaluate permeability, fluorescent particles with an average size of 0.03 μm (L5155, Sigma Aldrich) are used. 150 μL of culture medium containing fluorescent particles at a concentration of 10 μg/mL is injected into the top of the culture insert 24 h after the growth of HUVECs. Samples of 50 μL are collected from the lower wells at 5-min intervals for up to 30 min, and 50 μL of fresh culture medium is injected into the well chamber after each interval to prevent pressure build-up across the membranes. The fluorescence intensity of the collected samples is measured using a multi-label plate reader (Enspire, PerkinElmer) at an excitation wavelength of 450 nm and an emission wavelength of 505.8 nm. Figure 6 illustrates the change in fluorescence intensity over time.

The results confirm that the fluorescent particles diffused into the lower well chamber as time progressed, with fluorescence intensity initially increasing before reaching a steady state (Fig. 6). These findings demonstrate that the membrane supports material exchange while culturing cells, functioning both as a cell-to-cell interface and a scaffold.

## Conclusion

In this study, we proposed a new method for fabricating porous membranes by spin-coating and curing liquid PDMS onto highly robust Ni micropillars fabricated using MEMS technology. Ni micropillars with dimensions commonly used in lung-on-a-chip systems (*D* = 8 μm, *P* = 30 μm; *D* = 10 μm, *P* = 20 μm) were fabricated, and the subsequent porous membranes were also produced successfully. These membranes were integrated with conventional cell culture inserts, and cell viability assays were conducted by culturing HUVECs. The viability ratios were 75.8% for the PDMS membranes and 80.9% for the conventional culture inserts, indicating no significant difference in cell viability between the two. Permeability assays performed on membranes cultured with HUVECs demonstrated that the porous membranes could effectively exchange materials during cell culture. These results confirm that the membranes fabricated by our proposed method are effective as culture membranes. This method enables the low-cost production of flexible, porous PDMS membranes, and so can contribute to the mass production of “organ-on-a-chip” devices, where the impact of tissue deformation on material permeability is important, such as in the infection process of virus particles in alveoli or the absorption process of micro-nanoplastics in the small intestine.

## Data Availability

The data supporting the findings of this study are available from the corresponding author upon reasonable request.
